# Is ABO Incompatible Living Donor Kidney Transplantation in Children a Better Option than the Use of Optimal Grafts From Deceased Donors? A Plea for Better Prioritization of Deceased Kidney Grafts for Children

**DOI:** 10.3389/ti.2023.11911

**Published:** 2023-09-18

**Authors:** Olivia Boyer, Lars Pape

**Affiliations:** ^1^ Néphrologie Pédiatrique, Centre de Référence des Maladies Rénales Héréditaires de l’Enfant et l’Adulte, Hôpital Necker - Enfants Malades, Assistance Publique - Hôpitaux de Paris, INSERM U1163, Institut Imagine, Université Paris Cité, Paris, France; ^2^ Department of Pediatric Nephrology, Children’s Hospital, University Hospital of Essen, University of Duisburg-Essen, Essen, Germany

**Keywords:** kidney, transplantation, graft, ABO incompatible, paediatric kidney transplantation, children, childhood

While we acknowledge that ABOi LDKTx can be successfully performed in children and has the advantage of reducing the waiting time and risks associated with prolonged dialysis whilst conferring the benefits of living donor transplantation [1], we would like to balance the conclusions made by the authors and outline valuable alternatives.

ABOi kidney transplantation carries a higher risk of rejection compared to ABO compatible transplantation, most particularly antibody-mediated rejection [2]. To overcome this, both extensive pre-transplant conditioning and additional pre-transplant immunosuppressive therapy are required and include desensitization techniques such as antigen-specific immunoadsorption, B cell-depleting monoclonal antibodies (mainly rituximab), and intensified immunosuppression protocols. Such complex treatments expose children to a higher risk of bacterial and viral infections [2], post-transplant lymphoproliferative disease, and other neoplasias. Apheresis techniques require central venous lines in the absence of an arteriovenous fistula, especially in children on peritoneal dialysis or with pre-emptive transplantation, and these procedures can be complicated by infection, thrombosis, or bleeding, and so jeopardize future access to dialysis. In addition, these techniques may be impractical or risky in young children due to the extracorporeal volume required during immunoadsorption sessions. ABO incompatible kidney transplantation is therefore rarely performed in children who weigh <20 kg.

From an economic standpoint, ABOi transplantation is more expensive and resource intensive than ABO compatible transplantation. Additional procedures, prolonged hospital stays, and specialized therapies required for desensitization significantly increase the overall cost of the transplant procedure. For this reason, it may not be available in every health framework. On the other hand, shorter dialysis times obviously spare costs. Moreover, living organ donation can have a financial impact on the donor and his or her family, depending on specific national policies and social security requirements.

Additionally, some parents may want to reserve the option of donating their kidney for a second transplant in adulthood, at an age when organ shortages can be even greater.

Furthermore, while transplants from living donors generally have a better prognosis than transplants from deceased donors, it should be noted that parents who are candidates for donation are increasingly older and have more co-morbidities [3], whereas children often receive transplants from young deceased donors whose parenchyma is generally well preserved at the time of donation. This may partly reduce the advantages of living donation in pediatric kidney transplantation.

We would therefore like to discuss alternatives to ABOi LDKTx in children.

Firstly, we call for better prioritization of the allocation of deceased donor kidney transplants in children who will eventually require several transplants over the course of a lifetime. Priority rules should include age-matching criteria that could guarantee prioritization of pediatric recipients for optimal transplants with shorter waiting times. The allocation policies for transplants vary between jurisdictions and healthcare systems. In France, for instance, absolute national priority is given to recipients under the age of 18 years for the two kidneys of any donor under the age of 18 [4]. Pediatric recipients are also given priority for one of the kidney transplants from donors aged between 18 and 29, in the absence of a recipient benefiting from a priority due to immunization or a multi-organ transplant. Pediatric priority is extended until the transplant if the candidate was under 18 at the start of dialysis. Similarly, in the United States, recipients younger than 18 have priority over donors under 35 years of age [5]. Spain, Italy, and Switzerland also have strong pediatric prioritization with short waiting times. However, this priority is more limited elsewhere, particularly in the Euro Transplant zone (comprising Holland, Belgium, Luxembourg, Germany, Austria, Croatia, and Slovenia) where it should be improved, as it is currently restricted to kidneys from donors aged under 18, who are allocated as a priority to recipients who are also younger than 18. The impact on waiting times for adults based on better pediatric prioritization would be very small because of the large difference in numbers on waiting lists. Moreover, the prioritization criteria would be regularly evaluated and refined to ensure equity, fairness, and transparency.

We agree with the authors that paired kidney exchange programs, also known as kidney swaps or paired donation, such as that in the United States, can be a good strategy for children. We are pleased to note that such a program has been initiated in the United Kingdom, and we hope that this will also be the case for other pediatric kidney transplant programs. Altruistic donation to children could also be allowed. We find it extremely difficult to understand why there is still so much political reluctance, particularly in countries like Germany and France.

Finally, the use of infant kidneys transplanted *en-bloc* in specialized centers may be an interesting alternative for reducing the waiting time for children on the list. Various series have shown good results with this strategy in specialized teams [6–9]. One retrospective study, for example, compared 72 children who had received an *en-bloc* kidney with 75 who had received a kidney from a living donor. The estimated glomerular filtration rate was significantly higher in children who had received an *en-bloc* kidney from the 5th to the 17th year after transplantation and the 25 years graft survival was similar in both groups [10]. Another option is the split of infant *en-bloc* kidneys and the allocation to two small pediatric recipients. This approach has been successful in specialized centers [11], and further increases the number of recipients.

Also of note, pediatric organ donation, which decreased significantly during the COVID-19 pandemic, as was also the case with adults, should be an absolute priority, and the rate of organ donation refusals must be reduced [12].

To conclude, it is essential to consider the advantages and disadvantages outlined above in the context of each child’s specific medical condition and individual circumstances. The decision to pursue ABOi LDKTx should be made in consultation with the child’s medical team, weighing the potential benefits against the associated risks. Regardless, pediatric organ donation must be promoted, and priority given to optimal kidneys for pediatric recipients, who will often undergo several kidney transplants in the course of their lives. This is described in detail in the position statement of the International Pediatric Transplant Association [13], which emphasizes the special obligations society has towards children, the fair innings argument, and cumulative and time-sensitive accrual of developmental morbidity.

## References

[1] Stojanovic J Paessler A. Considering ABO Incompatible Living Donor Kidney Transplantation Before Deceased Donor Kidney Transplantation in Children: A Letter to the Editor. Transplant Int. 36:11613. 10.3389/ti.2023.11613 PMC10542673 37789915

[2] Cozzi M Donato P Ugolini G Nguefouet Momo RE Nacchia F Ballarini Z Outcomes in AB0 Incompatible Living Donor Kidney Transplantation: A Case - Control Study. Front Med (Lausanne) (2022) 9:932171. 10.3389/fmed.2022.932171 35935799 PMC9353324

[3] Qiu J Wang C Liang X Chen G Huang G Fu Q Effect of Donor Age and Parent-To-Child Transplant on Living-Related Donor Kidney Transplantation: A Single Center’s Experience of 236 Cases. Ren Fail (2015) 37:1007–12. 10.3109/0886022X.2015.1052948 26042341

[4] Macher M-A. La Transplantation Rénale Pédiatrique. Réanimation (2014) 23:690–7. 10.1007/s13546-014-0933-6

[5] Hippen BE Thistlethwaite JR Ross LF. Risk, Prognosis, and Unintended Consequences in Kidney Allocation. N Engl J Med (2011) 364:1285–7. 10.1056/NEJMp1102583 21410392

[6] Kizilbash SJ Evans MD Chinnakotla S Chavers BM. Survival Benefit of *En Bloc* Transplantation of Small Pediatric Kidneys in Children. Transplantation (2020) 104:2435–43. 10.1097/TP.0000000000003158 32022736 PMC7604907

[7] Laube GF Kellenberger CJ Kemper MJ Weber M Neuhaus TJ. Transplantation of Infant *en Bloc* kidneys Into Paediatric Recipients. Pediatr Nephrol (2006) 21:408–12. 10.1007/s00467-005-2129-9 16382315

[8] Afanetti M Niaudet P Niel O Faust MS Cochat P Berard E. Pediatric *en Bloc* Kidney Transplantation Into Pediatric Recipients: The French experience. Pediatr Transpl (2012) 16:183–6. 10.1111/j.1399-3046.2012.01654.x 22360402

[9] Beetz O Weigle CA Nogly R Klempnauer J Pape L Richter N Surgical Complications in Pediatric Kidney Transplantation—Incidence, Risk Factors, and Effects on Graft Survival: A Retrospective Single‐Center Study. Pediatr Transpl (2021) 25:e13871. 10.1111/petr.13871 33053269

[10] Sureshkumar KK Habbach A Tang A Chopra B. Long-Term Outcomes of Pediatric *En Bloc* Compared to Living Donor Kidney Transplantation: A Single-Center Experience With 25 Years Follow-Up. Transplantation (2018) 102:e245–8. 10.1097/TP.0000000000002104 29346254

[11] Hoyer DP Dittmann S Büscher A Benkö T Treckmann JW Gallinat A Kidney Transplantation With Allografts From Infant Donors-Small Organs, Big Value. Pediatr Transpl (2020) 24:e13794. 10.1111/petr.13794 32757309

[12] Putzer G Gasteiger L Mathis S van Enckevort A Hell T Resch T Solid Organ Donation and Transplantation Activity in the Eurotransplant Area During the First Year of COVID-19. Transplantation (2022) 106:1450–4. 10.1097/TP.0000000000004158 35411875 PMC9213062

[13] Freeman MA Botha J Brewer E Damian M Ettenger R Gambetta K International Pediatric Transplant Association (IPTA) Position Statement Supporting Prioritizing Pediatric Recipients for Deceased Donor Organ Allocation. Pediatr Transpl (2023) 27(1):e14358. 10.1111/petr.14358 36468303

